# Time efficiency and reliability of established computed tomographic obstruction scores in patients with acute pulmonary embolism

**DOI:** 10.1371/journal.pone.0260802

**Published:** 2021-12-03

**Authors:** Hans-Jonas Meyer, Nikolaos Bailis, Alexey Surov

**Affiliations:** 1 Department of Diagnostic and Interventional Radiology, University of Leipzig, Leipzig, Germany; 2 Department of Radiology and Nuclear medicine, University of Magdeburg, Magdeburg, Germany; Humanitas Clinical and Research Center - IRRCS, ITALY

## Abstract

**Objective:**

Acute pulmonary embolism (PE) is a life-threatening disease with a high mortality. Computed tomographic pulmonary angiography (CTPA) is used in clinical routine for diagnosis of PE. Many pulmonary obstruction scores were proposed to aid in stratifying clinical course of PE. The purpose of the present study was to compare common pulmonary obstruction scores in PE in regard of time efficiency and interreader agreement based upon a representative patient sample.

**Methods:**

Overall, 50 patients with acute PE were included in this single center, retrospective analysis. Two readers scored the CT images blinded to each other and assessed the scores proposed by Mastora et al., Qanadli et al., Ghanima et al. and Kirchner et al. The required time was assessed of each reading for scoring.

**Results:**

For reader 1, Mastora score took the longest time duration, followed by Kirchner score, Qanadli score and finally Ghanima score (every test, p<0.0001). The interreader variability was excellent for all scores with no significant differences between them. In the Spearman’s correlation analysis strong correlations were identified between the scores of Mastora, Qanadli and Kirchner, whereas Ghanima score was only moderately correlated with the other scores. There was a weak correlation between time duration and Mastora score (r = 0.35, p = 0.014). For the Ghanima score, a significant inverse correlation was found (r = -0.67, p<0.0001).

**Conclusion:**

For the investigated obstruction scores, there are significant differences in regard of time consumption with no relevant differences in regard of interreader variability in patients with acute pulmonary embolism. Mastora score requires the most time effort, whereas the score by Ghanima the least time.

## Introduction

Acute pulmonary embolism (PE) is a possible life-threatening disease with 30‐day mortality rates ranging from 0.5% to over 20% depending on clinical symptoms at presentation [1]. However, there are also low-risk clinical courses without severe complications. Therefore, immediate risk stratification of patients with acute PE at the time of presentation is crucial for the planning of patient care.

Computed tomographic pulmonary angiography (CTPA) has been established as the diagnostic gold standard in the detection of PE [2, 3]. So, the sensitivity and specificity were reported in some studies to be up to 100% [2]. Since then, the CT technique has significantly improved, especially due to increasing CT slices and consequently better image quality.

In clinical routine, the radiologist assesses, whether there is the presence of PE or not. Yet, there are some CT signs, which harbor prognostic information to guide treatment planning and to predict mortality [4]. In clinical evaluation, the right ventricle to left ventricle (LV) diameter-ratio was identified to be the strongest predictive value and most robust to predict clinical outcomes in patients with acute PE [3]. The contrast media reflux into the inferior vena cava has been reported as a significant prognostic marker in acute PE [5, 6].

The quantified total embolus burden represents another important CTPA parameter. The rationale is that more obstructed vessels lead to higher resistance and therefore to right heart insufficiency. In fact, in the first studies, the scores were associated with invasive pulmonary angiography and were able to predict short-term mortality. For example, Wu et al. found that clot burden quantified on CT pulmonary angiography was an important predictor of death in patients with PE [7]. Similar results were also reported by Van der Meer et al. [8]. However, other authors did not find any associations between total clot burden and mortality in PE [9–11].

Despite the growing body of literature regarding embolus burden scores, there are only few comparisons between these scores [12]. Moreover, the complexity of the scores differs significantly. As such, for the score proposed by Mastora et al. [13] (every pulmonary vessel is scored from 0 to 5, reflecting no embolism with 0 and complete obstruction with 5 points, whereas for the score proposed by Ghanima et al. [14] only the level of the most proximal vessel obstruction is quantified. The resulting point range is 0 to 155 for Mastora score and 0 to 4 for Ghanima score. These could result in significant differences to reflect clinical features depending on the score employed.

In our clinical experience, the time effort to quantify these scores differs significantly. Some of the obstruction scores were rated as too cumbersome to perform in clinical routine [3]. Moreover, the interreader agreement might be higher for the simpler scores compared to the score by Mastora et al. Clearly, there is need to investigate the differences of these pulmonary embolism scores.

Therefore, the purpose of the present study was to compare four of the most commonly used pulmonary obstruction scores for PE in regarding time efficiency and interreader agreement based upon a representative patient sample.

## Methods

This retrospective study was approved by the institutional review board (Nr: 118/19-ck, Ethics Committee, University of Leipzig, Leipzig, Germany).

The patient sample was obtained from a larger study sample, which assessed the associations between Mastora score and clinical features in patients with acute PE [6, 11]. Inclusion criteria were sufficient pulmonary vessel contrast and a representative PE manifestation. Exclusion criteria were patients with only small subsegmental emboli. Patients with other pulmonary diseases, such as pneumonia, cardiac decompensation or pleural effusions, were not excluded to ensure external validity. The CT scans were obtained between 2015 and 2018.

There were overall 50 patients (24 females, 48%) with a mean age of 63.4 ±18.1 years, range 19–100 years.

### Imaging technique

CTPA was performed on a 128-slice CT scanner (Ingenuity 128, Philips, Hamburg, Germany). Intravenous administration of an iodine-based contrast medium (60 mL Imeron 400 MCT, Bracco Imaging Germany GmbH, Konstanz, Germany) was given at a rate of 4.0 mL/s via a peripheral venous line. Automatic bolus tracking was performed in the pulmonary trunk with a trigger of 100 Hounsfield units (HU). Typical imaging parameters were: 100 kVp; 125 mAs; slice thickness = 1 mm; and pitch = 0.9. CTPA was performed in every case in deep inspiration level.

### PE scores

#### Mastora score

This scoring system includes 5 mediastinal, 6 lobar, and 20 segmental arteries, which were each scored for the degree of luminal obliteration due to embolism on a scale from 0 to 5 (0 = 0%, 1 = 1–24%, 2 = 25–49%, 3 = 50–74%, 4 = 75–99%, 5 = 100%). The sum of mediastinal, lobar and segmental artery scores gives a global obstruction score with a maximum of 155 [13].

#### Qanadli score

This obstruction score (0–100%) was defined based on the number of obstructed segmental arteries and was corrected according to the estimated degree of occlusion of each vessel (1 for partial obstruction; 2 for complete obstruction) [14].

#### Ghanima score

This obstruction score is based on the proximal extension of the embolus relative to the main pulmonary arteries. The pulmonary arterial tree is divided into four levels, and the score is calculated according to the level of the proximal extension in each lung: mediastinal arteries (4 points), lobar arteries (3 points), segmental arteries (2 points), and subsegmental (1 point) [15].

#### Kirchner score (modified Miller score)

The fourth score was calculated as reported by Kirchner et al. [16], which is a modified score of a previous proposed by Miller et al. [17]. This score ranges from 0 to 16. The presence of embolic material is rated using a two-point scale (0 absent, 1 present) within a total of 16 segmental arteries.

### Image analysis and assessment of time duration

The images were evaluated by two advanced residents (NB, HJM) with 5 and 4 years of general radiological experience including CT imaging, respectively.

Pulmonary embolism is defined as contrast media filling defect within a pulmonary vessel at least on 2 slices.

Before the patient images were read, both radiologists were trained for 2 hours in scoring PE with these scores with images of other patients with PE. To reduce possible recognition bias, there was one week delay between the estimation of different scores in these cases.

The required time to assess the scores was calculated with a stopwatch. The start was defined with the opening of the CT study, the end with record of the score into a spreadsheet. Both readers were blinded to each other’s results.

**Fig 1:**
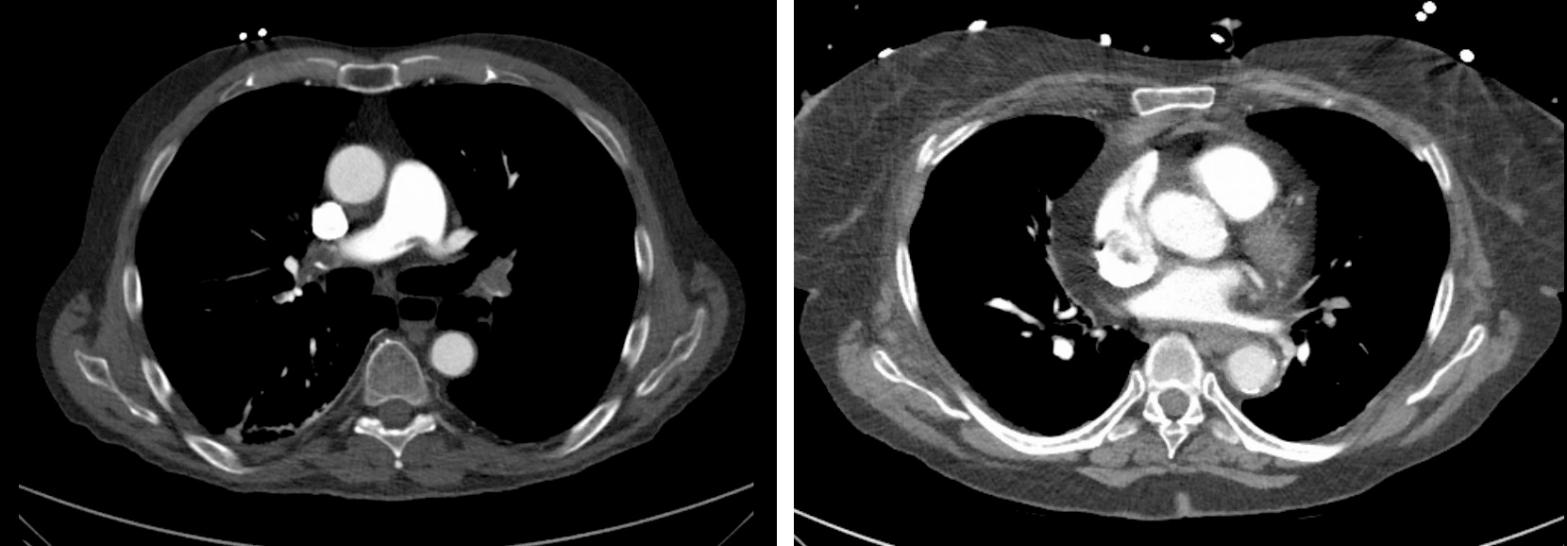
Representative patients of the patient sample. **a**. 79-years old male patient with massive acute pulmonary embolism. A saddle thrombus of the pulmonary trunk can be appreciated. The Mastora score was for reader 1 109, for reader 2 75, the Qanadli for reader 1 50, for reader 2 40, Ghanima score was 4 for both readers and Kirchner score was 12.25 for reader 1 and 11 for reader 2. The time duration was longest for Mastora score for both readers, 463 s and 281 s, followed by Qanadli 53 and 35 s, Kirchner 36 s and 167 s and the Ghanima with 13 and 6 s. **b**. 84-years old female patient with segmental acute pulmonary embolism of the lower lobes. The Mastora score was for reader 1 45, for reader 2 29, the Qanadli for reader 1 22.5, for reader 2 20, Ghanima score was 2 for both readers and Kirchner score was 3 for both readers. The time duration was longest for Mastora score for both readers, 187 s and 151 s, followed by Qanadli 150 and 34 s, Kirchner 92 s and 32 s and the Ghanima with 26 and 9 s.

Fig 1 provides CT images of representative patients of our sample for illustration purposes.

### Statistical analysis

The statistical analysis and graphics creation were performed using GraphPad Prism 5 (GraphPad Software, La Jolla, CA, USA). Collected data were evaluated by means of descriptive statistics (absolute and relative frequencies). Spearman’s correlation coefficient (r) was used to analyze associations between the investigated scores. Intraclass coefficient (ICC) was used to calculate interreader variability. Bland-Altman plots were used to visualize the interreader variability. Group differences were calculated with Mann-Whitney test. In all instances, p values <0.05 were taken to indicate statistical significance.

## Results

The descriptive score results are provided by Table 1.

**Table 1:** Overview of the pulmonary obstruction scores according to the reader.

Pulmonary obstruction score	Reader 1 mean ± SD, range	Reader 2 mean ± SD, range	Reader 1, Duration in s, mean ± SD, range	Reader 2, Duration in s, mean ± SD, range
**Mastora**	86.2 ±21.7, 35–121	80.8 ± 29.6, 21–132	311 ± 71.1, 142–463	185.2 ± 43.9, 96–286
**Qanadli**	48.2 ± 14.7, 12.5–82.5	43.6 ± 15.6, 12.5–80	80.6 ± 49.6, 9–180	48.0 ± 7.5, 32–72
**Ghanima**	3.2 ± 0.8, 1–4	3.4 ± 0.7, 1–4	26.2 ± 16.1, 10–69	7.3 ± 3.2, 4–20
**Kirchner**	9.9 ± 3.4, 2.3–14.8	9.5 ± 3.1, 3–14.5	162.4 ± 49.9, 53–270	28.5 ± 10.4, 15–75

Abbr. SD = standard deviation, s = second.

For reader 1, Mastora score took the longest time duration, followed by Kirchner score, Qanadli score and finally Ghanima score (every test p<0.0001).

For reader 2, Mastora score also took the longest time duration, followed by Qanadli score, Kirchner score and Ghanima score (every test p<0.0001).

**Fig 2:**
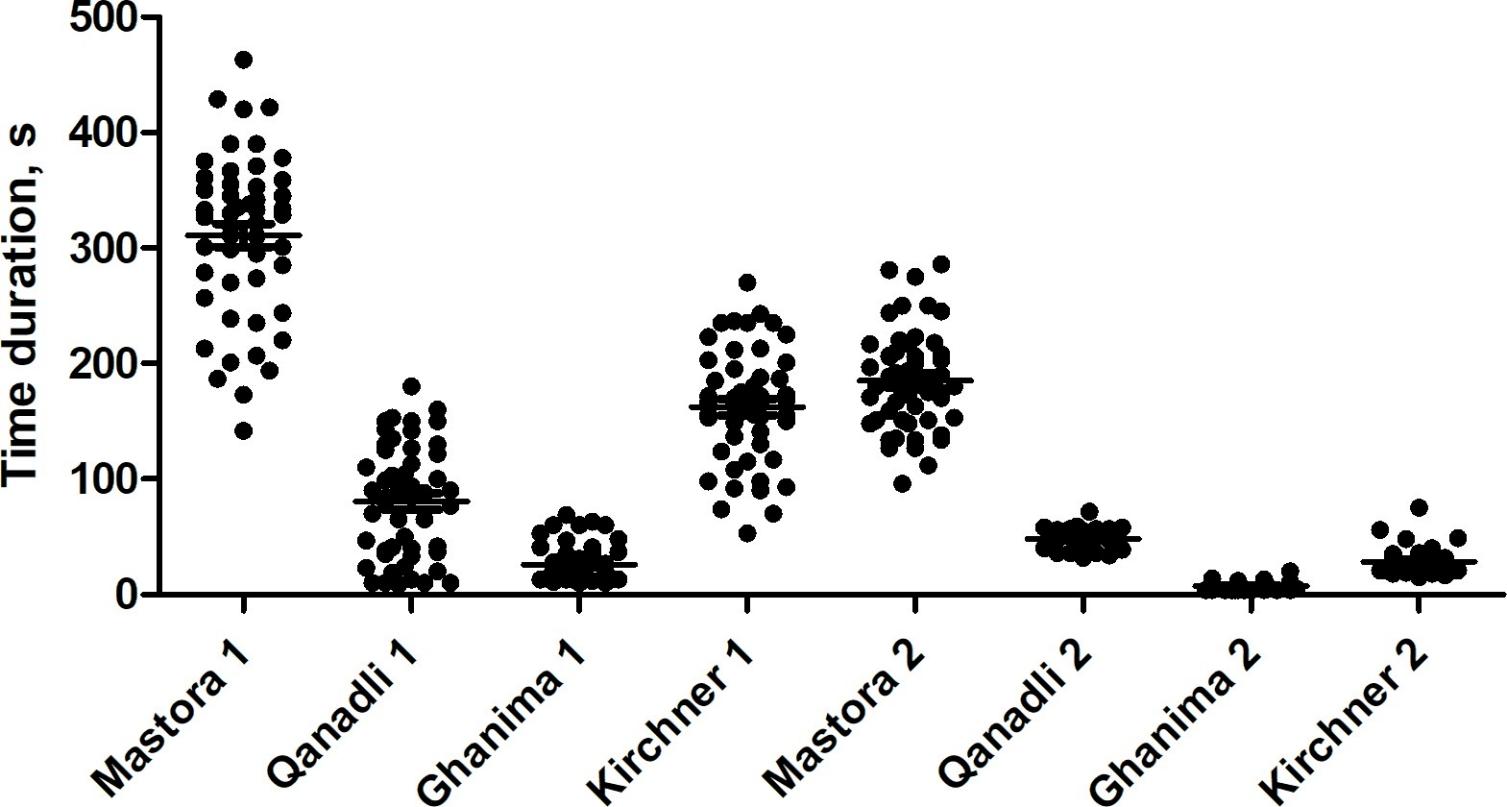
Scatter plot displaying the time duration for scoring in seconds (s). For reader 1, Mastora took the longest time duration, followed by Kirchner et al., Qanadli and finally Ghanima (every test p<0.0001). For reader 2, Mastora also took the longest time duration, followed by Qanadli, Kirchner and Ghanima (every test p<0.0001). There were significant differences between reader 1 and 2 in regard of time duration. So, reader 2 was significantly faster for every score (p<0.0001 for every score).

There were significant differences between reader 1 and 2 in regard of time duration. So, reader 2 was significantly faster for every score (p<0.0001 for every score). Fig 2 displays the time duration for every score as scatter plots.

**Fig 3:**
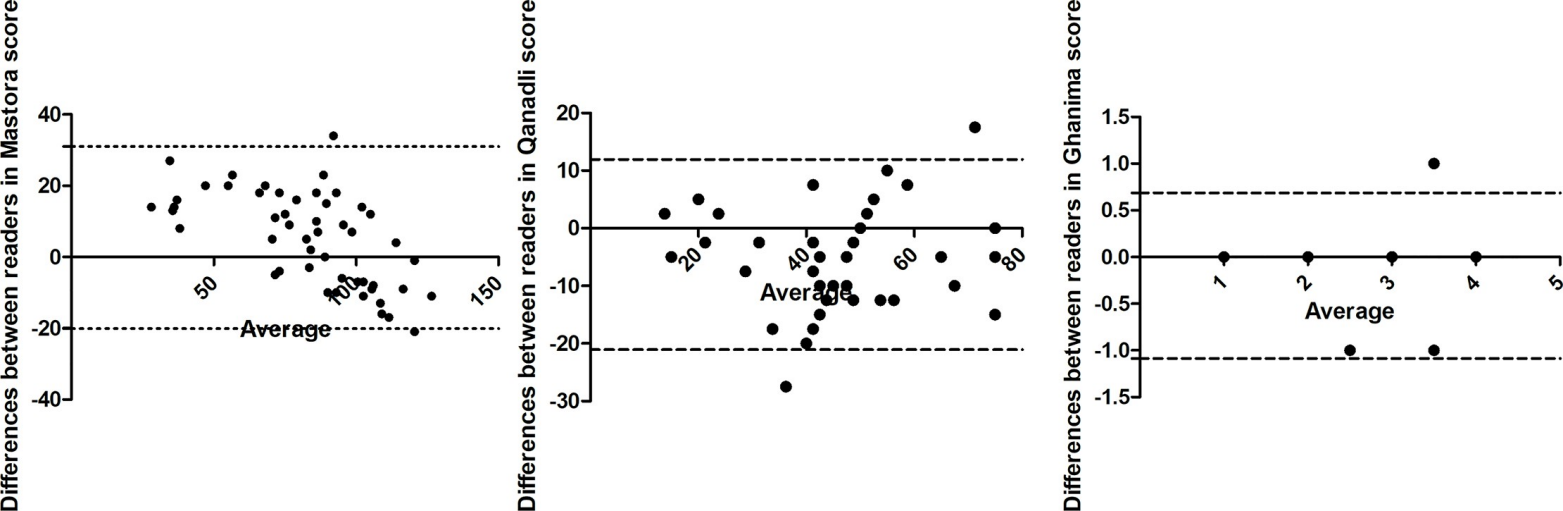
Bland-Altman plots displaying the excellent interreader variability of the scores. The dotted lines indicating the 95% agreement between the readers. A. The plot for Mastora score. B. The plot for Qanadli score. C. The plot for Ghanima score. D. The plot for Kirchner score.

Regarding the interreader variability, all scores showed excellent ICC values with no significant differences between them (Table 2, p>0.05). The results of the Bland-Altman plots are shown in Fig 3 to illustrate the interreader variability.

**Table 2:** Intraclass coefficients of the investigated pulmonary obstruction scores.

Pulmonary obstruction score	ICC
**Mastora**	0.87 95%CI 0.78–0.92
**Qanadli**	0.87 95% CI 0.74–0.90
**Ghanima**	0.81 95% CI 0.70–0.89
**Kirchner**	0.95 95% CI 0.93–0.97

Abbr. CI = Confidence interval, ICC = Intraclass coefficient.

In the Spearman’s correlation analysis, strong correlations were identified between Mastora score, Qanadli score and Kirchner score, whereas Ghanima score was only moderately correlated with the other scores (Table 3).

**Table 3:** Spearman’s correlation analysis of the investigated pulmonary obstruction scores.

Pulmonary obstruction score	Mastora	Qanadli	Ghanima
**Mastora**	x		
**Qanadli**	r = 0.76, p<0.0001	x	
**Ghanima**	r = 0.56, p<0.0001	r = 0.41, p = 0.002	x
**Kirchner**	r = 0.93, p<0.0001	r = 0.74, p<0.0001	r = 0.52, p<0.0001

**Fig 4:**
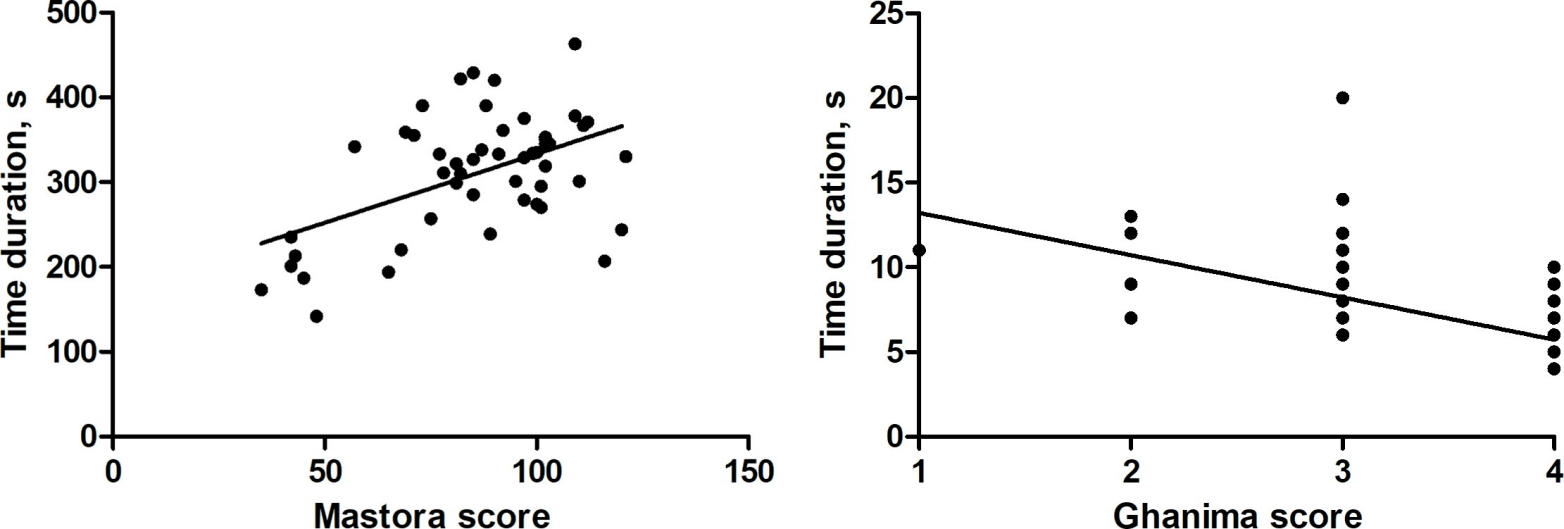
**A.** Spearman’s correlation analysis between time duration and the investigated scores. A weak positive correlation was identified with the Mastora score (r = 0.35, p = 0.014). **B.** A significant inverse correlation with the time duration was identified with the Ghanima score (r = -0.67, p<0.0001).

There was a weak correlation between the time duration and Mastora score (r = 0.35, p = 0.014). For Ghanima score, a significant inverse correlation with the time duration was identified (r = -0.67, p<0.0001, Fig 4). Regarding the other scores, no correlation was identified with time duration (Qanadli score r = -0.19, p = 0.19, Kirchner score r = 0.17, p = 0.24).

## Discussion

The present study investigated commonly used pulmonary embolism obstruction scores of their interreader variability and the time effort to score them. There were significant differences regarding time consumption between the scores. Notably, all of them had an excellent interreader agreement.

CT is the imaging modality of choice to diagnose and to rule out PE [2, 3]. There is extensive scientific effort to obtain potential biomarker from CT images and not only qualitatively assess the images by the radiologists.

With this approach, possible novel biomarkers could be obtained to predict possible complications and mortality in PE. This is especially of interest as PE is a potential life-threatening disease with a high mortality [1]. It could be crucial to predict early a possible hazardous course of PE, especially as CT imaging is often one of the first diagnostic procedures in these patients. There are several proposed imaging signs, which were identified to have prognostic implications [4]. Potential CT-signs were the ratio right ventricular diameter to left ventricular diameter and contrast media reflux into the inferior vena cava [4–6].

There is extensive body of literature which investigated the possible clinical benefit of embolism obstruction score [9]. Different scoring systems were proposed. As a common finding, all of them rated the location of the embolism with more proximal location in a higher rating. However, those scores were primarily validated by the authors of the studies and only few independent evaluations were performed.

The score by Mastora et al. is very complex, which scores the amount of obstruction of every pulmonary vessel up to the segmental vessels with 0 to 5 [13]. For the Ghanima score, the radiologist just has to locate the proximal embolism resulting in a score value from 0 to 4 [15].

These requirements of the scores result in different time effort to for the scoring. So, the score of Mastora needs the most time, followed by the scores of Qanadli and Kirchner, whereas the score of Ghanima was the fastest score. In clinical routine, Ghanima score can therefore easily be reported by the radiologist with only little effort.

Moreover, there is a weak correlation between the time duration and Mastora score indicating that more occluded pulmonary vessels result into more time to calculate the score. For the other scores, no similar association was identified, which can be interpreted that they are not dependent on overall embolus burden and complex patient cases are as fast to calculate as patients with less involved vessels.

On the other hand, a strong inverse correlation between the time duration and Ghanima score value was identified, which can be interpreted that severe, proximal occlusions are fast and easily to score. The radiologist just needs to scroll to the pulmonary trunk and the main pulmonary vessels to see a large embolism which results in a score of 4. This score can, therefore, be easily used in clinical routine.

For all scores the interrreader variability was very good to excellent with no substantial differences between the scores. These results are good comparable to the interreader agreement published in the study by Aribas et al. [12].

According to the literature, the scores of Mastora and Qanadli were most often used to assess the pulmonary obstruction and to correlate them with possible outcome predictors.

In other studies, these promising results could not be replicated. So, Bach et al. could not identify predictive power of Mastora score for 30 days mortality [5]. Similar results were reported also by other authors [6]. Moreover, Mastora score was only weakly correlated with lactate level and not with other serological or clinical parameters [11].

Contrary to other studies, some correlations with clot burden were reported. For example, Thieme et al. [18], reported a statistically significant correlation between Mastora score and troponin level (r *=* 0.37, *p* = 0.016). Furthermore, Gül et al. investigated 28 patients with PE and identified a slight correlation between Qanadli score and troponin level (*r =* 0.32, *p* = 0.01) [19]. Similarly, Jeebun et al. also showed a significant correlation between clot burden and troponin level (*r =* 0.41, *p* = 0.048) [20]. In brief, there are only weak correlations, if any, between clot burden and serological parameters in PE patients to assume.

Ghanima et al. proposed a simple score to predict right heart dilation and serum troponin levels [15]. Yet contrary to first believe, the proximal localization of the embolus is not associated with all-cause mortality, which could lead to a weaker prognostic relevance of this score [4]. This might be of interest, as the score by Ghanimi et al does not utilize differences regarding multiple emboli, which might fail to detect clinically relevant information. For example, a patient with multiple segmental emboli is scored the same as a patient with only one segmental embolus with a score value of 2.

One finding of the present study is that only moderate correlations between the other scores were identified with Ghanima score. This proposedly can indicate that the simplified approach by Ghanima also leads to loss of information which the other scores still harbor. Thus, in a recent study, Ghanima score showed the worst area under the curve to predict right ventricular dysfunction and was significantly lower compared to the other scores [12]. Undoubtedly, there is need for more research to assess, whether these differences also lead to differences in prediction of mortality as an outcome parameter in PE patients.

In a recent study, 30 patients with pulmonary embolism were investigated by human reading and by a computed aided approach to calculate the obstruction scores by Mastora and Qanadli [21]. A mean time value of 374.9 ± 150.2 s for calculation both scores was reported, which is good comparable with the present results. The authors reported a computed aided approach, which significantly reduces the time effort to calculate the scores. However, the study included only patients with a small embolus burden with a mean Mastora score of 20.5, which is significantly lower than in the present study. Presumably, patients with segmental embolism in only one lobe are easier and faster to score than patients with bilateral and severe embolism.

Nevertheless, in the future computed aided approaches and even machine learning techniques will help to further score patients with PE [22].

Possible reasons for the interreader variability are filling defects within the vessels, which may be misdiagnosed with an embolism. This could especially be of importance for the segmental and subsegmental level. Thus, scores utilizing smaller vessels with a higher weighting of this pulmonary level, mostly the score by Mastora et al., suffer from this limitation. Another one can be vessel anomalies which might mislead to diagnose lobar instead of segmental PE and in this way might have an influence of all scores, yet, again, especially of Mastora score.

A strength of the present study is that a CT scanner with 128 slices was used, which is a newer generation CT compared to the CT scanner technology used in the studies to first validate the proposed scores. Presumably, due to higher imaging quality and better contrast of the pulmonary vessel the assessment of small emboli is improved.

There are some limitations of the present study to address. Firstly, the scores were only assessed by advanced residents. Yet, they are experienced in CT imaging and were trained in the scoring before the study CTs were evaluated. The external validity for the analysis can be assumed as sufficient. Secondly, there might be recognition bias, after the first scoring of the patients. However, there was sufficient time between the reading sessions to reduce this possible bias.

In conclusion, there are significant differences in regard of time consumption with no relevant differences of interreader variability in obstruction scores for pulmonary embolism. Mastora score needs most time effort, whereas the score by Ghanima the least time. However, future studies are needed to assess, whether this simplified scoring method is as good for prediction of individual clinical course.

## Supporting information

S1 Table Pulmonary embolism scores. Study table of the investigated Pulmonary Embolism scores of the present analysis.
(XLSX)
